# Structural Complexity of DNA Sequence

**DOI:** 10.1155/2013/628036

**Published:** 2013-04-04

**Authors:** Cheng-Yuan Liou, Shen-Han Tseng, Wei-Chen Cheng, Huai-Ying Tsai

**Affiliations:** Department of Computer Science and Information Engineering, National Taiwan University, Taipei 10617, Taiwan

## Abstract

In modern bioinformatics, finding an efficient way to allocate sequence fragments with biological functions is an important issue. This paper presents a structural approach based on context-free grammars extracted from original DNA or protein sequences. This approach is radically different from all those statistical methods. Furthermore, this approach is compared with a topological entropy-based method for consistency and difference of the complexity results.

## 1. Introduction 

DNA sequence analysis becomes important part in modern molecular biology. DNA sequence is composed of four nucleotide bases—adenine (abbreviated A), cytosine (C), guanine (G), and thymine (T) in any order. With four different nucleotides, 2 nucleotides could only code for maximum of 4^2^ amino acids, but 3 nucleotides could code for a maximum 4^3^ amino acids. George Gamow was the first person to postulate that every three bases can translate to a single amino acid, called a codon. Marshall Nirenberg and Heinrich J. Matthaei were the first to elucidate the nature of a genetic code. A short DNA sequence can contain less genetic information, while lots of bases may contain much more genetic information, and any two nucleotides switch place may change the meaning of genetic messages.

Sequence arrangement can produce many different results, but only few codons exist in living bodies. Some sequences do not contain any information which is known as junk DNA. Finding an efficient way to analyze a sequence fragment corresponding to genetic functions is also a challenging problem.

In recent papers, methods broadly fall into two categories, sequence complexity [1, 2] and structural pattern analysis [3–8]. Koslicki [1] presented a method for computing sequence complexities. He redefined topological entropy function so that the complexity value will not converge toward zero for much longer sequences. With separate sequence into several segments, it can determine the segments where are exons or introns, and meaningful or meaningless. Hao et al. [7] given a graphical representation of DNA sequence, according to this paper, we can find some rare occurred subsequences. R. Zhang and C. T. Zhang [4] used four-nucleotide-related function drawing 3D curves graph to analyze the number of four-nucleotide occurrence probabilities. Liou et al. [9] had given a new idea in modeling complexity for music rhythms; this paper translated text messages into computable values, so computers can score for music rhythms.

In this paper, we propose a new method for calculating sequences different from other traditional methods. It holds not only statistical values but also structural information. We replace four nucleotides with tree structure presented in [9] and use mathematical tools to calculate complexity values of the sequences. So we can compare two sequences with values and determine dissimilarity between these two sequences. In biomedical section, we can use this technique to find the effective drugs for new virus with priority.

## 2. DNA Sequence Represented with Tree Structure

Our method uses Lindenmayer system [10–12] property among calculated complexities from tree structure [9]; it is a different way of computing complexities of sequences. At first, we introduce *DNA tree* and convert DNA sequence to tree structure. A DNA tree is a binary tree of which each subtree is also a DNA tree. Every tree node is either a terminal node or a node with two childrens (branches or descendants).

Lindenmayer system is a powerful rewriting system used to model the growth processes of plant development. We will introduce it in Section 2.2 in detail. Lindenmayer system uses some initial and rewriting rules to construct beautiful graphs. Since it can construct a tree from rewriting rules, it also can extract rewriting rules from a tree. In this section, we will use tools to generate the rules from tree.

**Figure 1:**
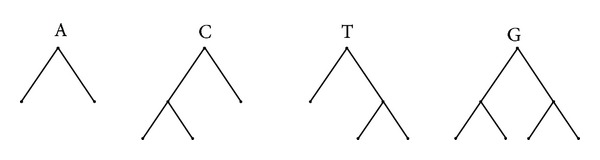
Nucleotide bases corresponding trees.

We use 4 fixed *tree representations* for nucleotide bases A, T, C, and G (see Figure 1). When we apply this method to amino acid sequence, we can construct more tree representation for amino acids, respectively.

**Figure 2:**
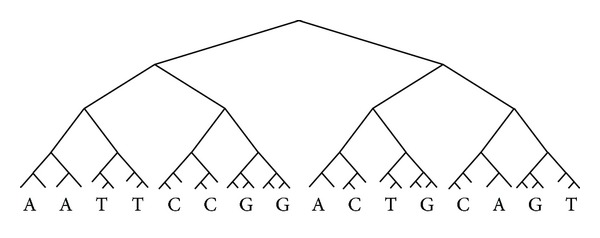
DNA sequence represented with tree structure.

When we transfer a sequence to DNA tree, we will replace every word to tree elements step by step, and two consecutive trees can combine to a bigger tree. Following the previous steps, a DNA sequence will be transfer to a DNA tree (see Figure 2).

### 2.1. Bracketed Strings for a DNA Sequence

For computing complexity of our DNA tree, we need some rules for converting tree to another structure. We use a stack similarly structure to represent the hierarchy of DNA tree, called *bracketed string*. DNA tree can transfer to a unique bracketed string by the following symbols, and it can transfer back to the original tree:

- *F*: the current location of tree nodes; it can be replaced by any word or be omitted;
- +: the following string will express the right subtree;
−: the following string will express the left subtree;
- [: this symbol is pairing with ]; “[⋯]” denotes a subtree where “⋯”; indicates all the bracketed strings of its subtree;
- ]: see [ description.

**Figure 3:**
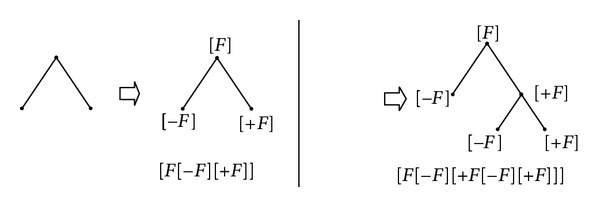
Bracketed strings representation for two trees.

Following the previous symbols, Figure 3 shows that nucleotide base A and T represented tree can transfer to [*F*[−*F*][+*F*]] and [*F*[−*F*][+*F*[−*F*][+*F*]]], respectively.

**Figure 4:**
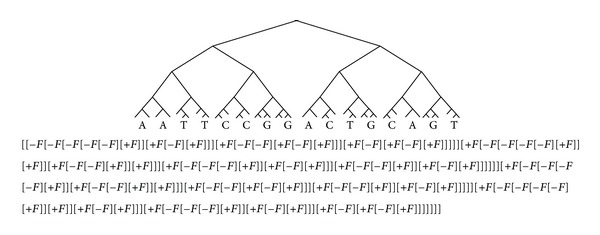
Bracketed strings representation for Figure 2.

**Figure 5:**
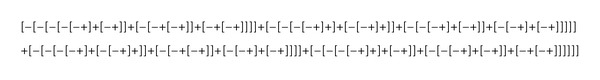
More simply bracketed strings representation for Figure 2.

And Figure 4 is the bracketed string of Figure 2. We can see that when the tree grows, string seems to be more redundant. Since we focus here only on DNA trees, we can simplify the bracketed string representations. First, our trees have only two subtrees. Second, the “*F*” notation for the tree is trivial. With these two characteristics, we may omit the “*F*” notation from the bracketed string and use only four symbols, {[, ], −, +}, to represent trees. In our cases, “[⋯]” denotes a subtree where “⋯” indicates all the bracketed strings of its subtrees. “−” indicated the next “[⋯]” notation for a tree is a left subtree of current node, and “+” is a right subtree vice versa.  Figure 5 is the simplified string of bracketed string shown in Figure 4.

### 2.2. DNA Sequence Represented with L-System

When we obtain DNA tree and bracketed string representation, we need rewriting rules for analyzing tree structure. There are some types of rewriting mechanism such as Chomsky grammar and Lindenmayer system (*L-system* for short). The largest difference between two string rewriting mechanisms lies in the technique used to apply productions. Chomsky grammar is suitable for applying productions sequentially, while L-system is for parallel. In our structure, applying L-system to our representations is better than Chomsky grammar.

The L-system was introduced by the biologist Lindenmayer in 1968 [13]. The central concept of the L-system is rewriting. In general, rewriting is a technique used to define complex objects by successively replacing parts of a simple initial object, using a set of rewriting rules or productions. In the next section, we will present how we use L-system to our DNA tree. The L-system is defined as follows.

**Definition 1**

L-system grammars are very similar to the Chomsky grammar, defined as a tuple [14]:

(1) $$\begin{array}{c} G=\left(V,\omega ,P\right), \end{array}$$

where

- *V* = {*s*_1_, *s*_2_,…, *s*_*n*_} is an alphabet,
- *ω* (start, axiom, or initiator) is a string of symbols from *V* defining the initial state of the system,
- *P* is defined by a production map *P* : *V* → V* with *s* → *P*(*s*) for each *s* in *V*. The identity production *s* → *s* is assumed. These symbols are called constants or terminals.

### 2.3. Rewriting Rules for DNA Sequences

**Figure 6:**
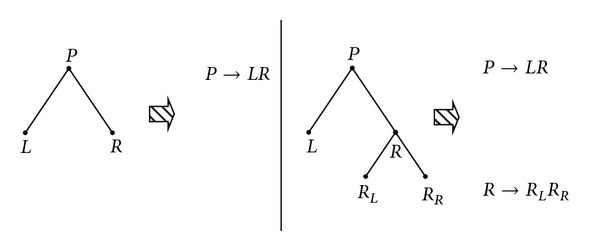
Example of rewriting rules for trees.

As discussed earlier, we want to generate the rules from DNA trees. In this section, we will explain how we apply rewriting rules to those trees. We can apply distinct variables to each node. Since the technique described previously always generates two subtrees for each node, for every nonterminal node, they always can be explained in the following format:

(2) $$\begin{array}{c} P\to LR, \end{array}$$

where *P* denotes the current node, *L* denotes its left subtree, and *R* denotes its right subtree, respectively. We give an example shown in Figure 6; left tree has three nodes and only root is nonterminal node, it can be rewritten as *P* → *LR*. Right tree has five nodes, root *P* with left subtree *L* and right subtree *R*. Left subtree is terminal, but right is not. *R* has two terminal subtrees *R*_*L*_ and *R*_*R*_, so this tree can be rewritten as *P* → *LR* and *R* → *R*_*L*_ *R*_*R*_.

### 2.4. Rewriting Rules for Bracketed Strings

Similarly, we can also use rewriting rules to generate bracketed strings. In rewriting rules for DNA trees shown in Section 2.3, we write *P* → *LR* for a tree with left and right subtrees. Note that we call *L* and *R* as the nonterminals. In this section, terminal nodes will be separated from trees, and we use “null” to represent a terminal. Such tree will have a corresponding bracketed string as follows: [[−*F*⋯][+*F*⋯]]. “[−*F*⋯]” represents the left subtree, while “[+*F*⋯]” represents the right subtree. Therefore, we can replace the rewriting rules with

**Figure 7:**
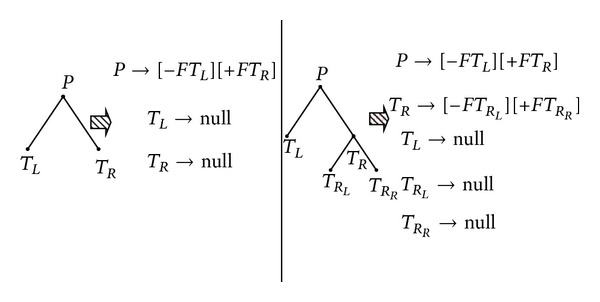
Rewriting rules for the bracketed string of trees.

(3) P→[−FL][+FR],F→⋯,R→⋯,

where “⋯” is the rewriting rule for the bracketed string of each subtree. For the sake of readability, we replace the words such as “*R*_*R*_*L*__” and “*R*_*R*_*R*__”. In Figure 7, we show the rewriting rules for the bracketed string of the tree in Figure 3.

**Figure 8:**
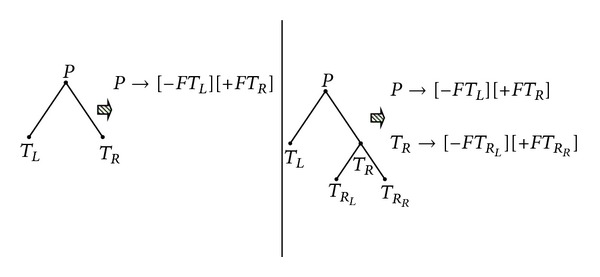
Rewriting rules for the bracketed string without nulls of trees.

As we can see, there are “nulls" in the rules. Those “nulls” do not have significant effects to our algorithm, so we simply ignore the nulls. Now, Figure 3 can apply new rewriting rules without trivial nulls as Figure 8.

When tree grows up, the rewriting rules may generate identical rules. Assume that we have the following rules:

(4) P→[−FTL][+FTR],TL→[−F][+F],TR→[−F][+FTRR],TRR→[−F][+FTRRR],TRRR→[−F].

These rules can generate exactly one bracketed string and, thus, exactly one DNA tree. All these rules form a rule set that represents a unique DNA tree. When we look at *T*_*R*_ → [−*F*][+*FT*_*R*_*R*__] and *T*_*R*_*R*__ → [−*F*][+*FT*_*R*_*R*_*R*___], they have the same structure since they both have a right subtree and do not have a left subtree. The only difference is that one of the subtrees is *T*_*R*_*R*__ and that the other is *T*_*R*_*R*_*R*___. We will define two terms to express the similarity between two rewriting rules, and these terms can simplify complexity analysis.

### 2.5. Homomorphism and Isomorphism of Rewriting Rules

At the end of the previous section, we discussed that *T*_*R*_ → [−*F*][+*FT*_*R*_*R*__] and *T*_*R*_*R*__ → [−*F*][+*FT*_*R*_*R*_*R*___] are almost the same. How can we summarize or organize an effective feature to them? Liou et al. [9] gave two definitions to classify similar rewriting rules described before as follows.

**Definition 2**

Homomorphism in rewriting rules. We define that rewriting rule *R*_1_ and rewriting rule *R*_2_ are homomorphic to each other if and only if they have the same structure.

In detail, rewriting rule *R*_1_ and rewriting rule *R*_2_ in DNA trees both have subtrees in corresponding positions or both not. Ignoring all nonterminals, if rule *R*_1_ and rule *R*_2_ generate the same bracketed string, then they are homomorphic by definition.

**Definition 3**

Isomorphism on level *X* in rewriting rules. Rewriting rule *R*_1_ and rewriting rule *R*_2_ are isomorphic on depth *X* if they are homomorphic and their nonterminals are relatively isomorphic on depth *X* − 1. Isomorphic on level 0 indicates homomorphism.

Applying to the bracketed string, we ignore all nonterminals in (4) as follows:

(5) P→[−FTL][+FTR]→[−F][+F],TL→[−F][+F]→[−F][+F],TR→[−F][+FTRR]→[−F][+F],TRR→[−F][+FTRRR]→[−F][+F],TRRR→[−F]→[−F].

We find that *P*, *T*_*L*_, *T*_*R*_, and *T*_*R*_*R*__ are homomorphic to each other; they generate the same bracketed string, [−*F*][+*F*]. But *T*_*R*_*R*_*R*___ is not homomorphic to any of the other rules; its bracketed string is [−*F*].

**Figure 9:**
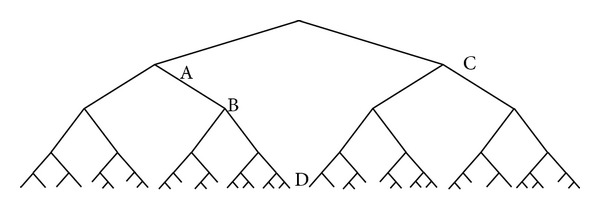
Example of homomorphism and isomorphism.

Let us recall DNA tree example in Figure 2; we will use this figure as an example to clarify these definitions. Now we marked some nodes shown in Figure 9; there are tree rooted at A, B, C, and D, respectively, tree A, tree B, tree C, and tree D. Tree A is isomorphic to tree C on depth 0 to 3, but they are not isomorphic on depth 4. Tree B is isomorphic to tree C on depth from 0 to 2, but they are not isomorphic on depth 3. D is not isomorphic to any other trees, nor is it homomorphic to any other trees.

After we define the similarity between rules by homomorphism and isomorphism, we can classify all the rules into different subsets, and every subset has the same similarity relation. Now we list all the rewriting rules of Figure 2 into Table 1 but ignore terminal rules such as “→ null” and transfer rule's name to class name (or class number). For example, we can give terminal rewriting rule a class, “*C*_3_→ null”, and a rule link to two terminals; we can give them “*C*_2_ → *C*_3_ *C*_3_"; here *C*_3_ is the terminal class. After performing classification, we obtain not only a new rewriting rule set but also a context-free grammar, which can be converted to automata.

In Table 1, rules such as *T*_*R*_*L*_*L*_*L*____ → [−*F*][+*F*], and *T*_*R*_*R*_*R*_*L*_*L*_____ → [−*F*][+*F*] and *T*_*R*_*L*_*R*_*L*_*R*_____ → [−*F*][+*F*] are isomorphic on depth 1 and assigned to Class 4. There are twenty such rules before classification, so we write “(20)*C*_4_ → [−*F*][+*F*]”. Similar rules such as *P* → [−*FT*_*L*_][+*FT*_*R*_], *T*_*R*_*L*_*L*_*L*____ → [−*F*][+*F*], and *T*_*R*_*R*_*R*_*R*____ → [−*F*][+*FT*_*R*_*R*_*R*_*R*_*R*_____] are isomorphic on depth 0, and there are 47 such rules. They are all assigned to Class 1 by following a similar classification procedure. The classification of the all rules is listed in Table 2. Note that this section also presents a new way to convert a context-sensitive grammar to a context-free one.

## 3. DNA Sequence Complexity

When we transfer the DNA sequence to the rewriting rules, and classify all those rules we attempt to explore the redundancy in the tree that will be the base for building the cognitive map [15]. We compute the complexity of the tree which those classified rules represent. We know that a classified rewriting rule set is also a context-free grammar, so there are some methods for computing complexity of rewriting rule as follows.

**Definition 4**

Topological entropy of a context-free grammar. The topological entropy *K*_0_ of (context-free grammar) CFG can be evaluated by means of the following three procedures [16, 17].

(1) For each variable *V*_*i*_ with productions (in Greibach form),
  (6) $$\begin{array}{c} V_{i}\to t_{i_{1}}U_{i_{1}},t_{i_{2}}U_{i_{2}},\ldots ,t_{i_{k_{i}}}U_{i_{k_{i}}}, \end{array}$$
  where {*t*_*i*_1__, *t*_*i*_2__,…, *t*_*i*_*k*_*i*___} are terminals and {*U*_*i*_1__, *U*_*i*_2__,…, *U*_*i*_*k*_*i*___} are nonterminals. The formal algebraic expression for each variable is
  (7) $$\begin{array}{c} V_{i}=\sum_{j=1}^{k_{i}}t_{i_{j}}U_{i_{j}}. \end{array}$$
(2) By replacing every terminal *t*_*i*_*j*__ with an auxiliary variable *z*, one obtains the generating function
  (8) $$\begin{array}{c} V_{i}\left(z\right)=\sum_{n=1}^{\infty }N_{i}\left(n\right)z^{n}, \end{array}$$
  where *N*_*i*_(*n*) is the number of words of length *n* descending from *V*_*i*_.
(3) Let *N*(*n*) be the largest one of *N*_*i*_(*n*), *N*(*n*) = max⁡{*N*_*i*_(*n*), for all *i*}. The previous series converges when *z* < *R* = *e* ^−*K*_0_^. The topological entropy is given by the radius of convergence *R* as
  (9) $$\begin{array}{c} K_{0}=-\ln R. \end{array}$$

Our productions have some difference from the aforementioned definitions. First, our productions are written in Chomsky-reduced form instead of Greibach form. Second, DNA is finite sequence; it generates finite tree, but the previous formulas are applied on infinite sequences. For convenience in the DNA tree case, we rewrite the definition as follows [9].

**Definition 5**

Topological entropy of context free grammar for DNA tree.

(1) Assume that there are *n* classes of rules and that each class *C*_*i*_ contains *n*_*i*_ rules. Let *V*_*i*_ ∈ {*C*_1_, *C*_2_,…, *C*_*n*_}, *U*_*ij*_ ∈ {*R*_*ij*_, *i* = 1,2,…, *n*, *j* = 1,2,…, *n*_*i*_}, and *a*_*ijk*_ ∈ {*x* : *x* = 1,2,…, *n*}, where each *U*_*ij*_ has the following form:
  (10) Ui1→Vai11Vai12,Ui2→Vai21Vai22,⋯→⋯,Uini→Vaini1Vaini2.
(2) The generating function of *V*_*i*_, *V*_*i*_(*z*) has a new form as follows:
  (11) $$\begin{array}{c} V_{i}\left(z\right)=\frac{\sum_{p=1}^{n_{i}}n_{ip}zV_{a_{ip1}}\left(z\right)V_{a_{ip2}}\left(z\right)}{\sum_{q=1}^{n_{i}}n_{iq}}. \end{array}$$
  If *V*_*i*_ does not have any nonterminal variables, we set *V*_*i*_(*z*) = 1.
(3) After formulating the generating function *V*_*i*_(*z*), we intend to find the largest value of *z*, *z* ^max⁡^, at which *V*_1_(*z* ^max⁡^) converges. Note that we use *V*_1_ to denote the rule for the root node of the DNA tree. After obtaining the largest value, *z* ^max⁡^, of *V*_1_(*z*), we set *R* = *z* ^max⁡^, the radius of convergence of *V*_1_(*z*). We define the complexity of the DNA tree as
  (12) $$\begin{array}{c} K_{0}=-\ln R. \end{array}$$

Now we can do some examples of computation procedure for the complexity. According to our definition, the given values for the class parameters are listed in Table 3. There are five classes, so we obtain the formulas for *V*_5_(*z*′), *V*_4_(*z*′), *V*_3_(*z*′), *V*_2_(*z*′), and *V*_1_(*z*′) successively. They are

(13) V5(z′)=1(by definition),V4(z′)=∑p=1n4n4pz′Va4p1(z′)Va4p2(z′)∑q=1niniq   =z′×(20×V5(z′)×V5(z′))20=z′,V3(z′)=∑p=1n3n3pz′Va3p1(z′)Va3p2(z′)∑q=1niniq   =z′×(4×V5(z′)×V4(z′))4=z′2,V2(z′)=∑p=1n2n2pz′Va2p1(z′)Va2p2(z′)∑q=1niniq   =z′×(4×V4(z′)×V5(z′))4=z′2,V1(z′)=∑p=1n1n1pz′Va1p1(z′)Va1p2(z′)∑q=1niniq   =8z′×V1(z′)2+2(z′)3×V1(z′)19    +(2(z′)5+2(z′)4+5(z′)3)19.

Rearranging the previous equation for *V*_1_(*z*′), we obtain a quadratic for *V*_1_(*z*′):

(14) 819(z′)×V1(z′)+(1−219(z′)3)×V1(z′)  +119(2(z′)5+2(z′)4+5(z′)3)=0.

Solving *V*_1_(*z*′), we obtain the formula

(15) $$\begin{array}{c} V_{1}\left(z^{\prime }\right)=\left(\frac{{\left(z^{\prime }\right)}^{2}}{4}-\frac{19}{8z^{\prime }}\right)\pm \frac{19}{8z^{\prime }}\sqrt{B^{2}-A}, \end{array}$$

where

(16) $$\begin{array}{c} A=\frac{32}{361}\left(2{\left(z^{\prime }\right)}^{6}+2{\left(z^{\prime }\right)}^{5}+5{\left(z^{\prime }\right)}^{4}\right),\\ B=1-\frac{2}{19}{\left(z^{\prime }\right)}^{3}. \end{array}$$

Finally, the radius of convergence, *R*, and complexity, *K*_0_ = −ln⁡*R*, can be obtained from this formula. But, computing the *z* ^max⁡^ directly is difficult, so we use iterations and region tests to approximate the complexity; details are as follows.

(1) Rewrite the generating function as
  (17) $$\begin{array}{c} V_{i}^{m}\left(z^{\prime }\right)=\frac{\sum_{p=1}^{n_{i}}n_{ip}z^{\prime }V_{a_{ip1}}^{m-1}\left(z^{\prime }\right)V_{a_{ip2}}^{m-1}\left(z^{\prime }\right)}{\sum_{q=1}^{n_{i}}n_{iq}},\\ V_{i}^{0}\left(z^{\prime }\right)=1. \end{array}$$
(2) The value from *V*_*i*_^0^(*z*′) to *V*_*i*_^*m*^(*z*′). When *V*_*i*_^*m*−1^(*z*′) = *V*_*i*_^*m*^(*z*′) for all rules, we say that *V*_*i*_^*m*^(*z*′) reach the convergence, but *z*′ is not the *z* ^max⁡^ we want. Here, we set *m* = 1000 for each iteration.
(3) Now we can test whether *V*_*i*_(*z*′) is convergent or divergent at a number *z*′. We use binary search to test every real number between 0 and 1; in every test, when *V*_*i*_(*z*′) converges, we set bigger *z*′ next time, but when *V*_*i*_(*z*′) diverges, we set smaller *z*′ next time. Running more iterations will obtain more precise radius.

## 4. Results

**Figure 10:**
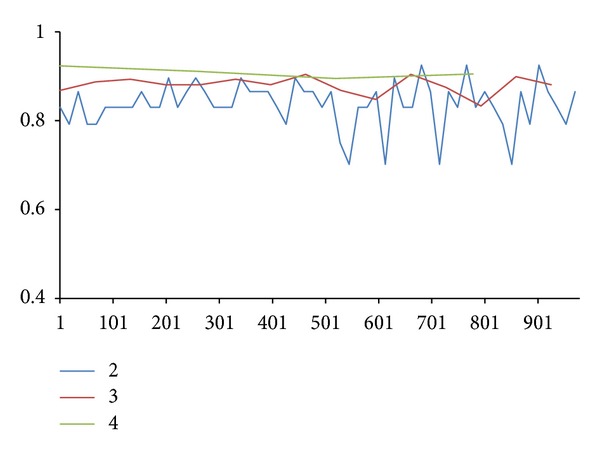
Koslicki method (topological entropy method, TE for short) example.

In 2011, Koslicki [1] gave an efficient way to compute the topological entropy of DNA sequence. He used fixed length depending on subword size to compute topological entropy of sequence. For example, in Figure 10 (all DNA and amino acid data can be found in NCBI website, http://www.ncbi.nlm.nih.gov/), the sequence length is 1027 characters, and there are three subword sizes 2, 3, and 4 with blue, red, and green lines, respectively. For larger subword size, much larger fragment is required for complexity computation. The required fragment size grows exponentially, while the length of sequence is not dependent on the growth rate of subword size, so it is not a good method for us overall.

We present a new method called structural complexity in previous sections, and there are several benefits from using our method instead of Koslicki method, described as follows.

**Figure 11:**
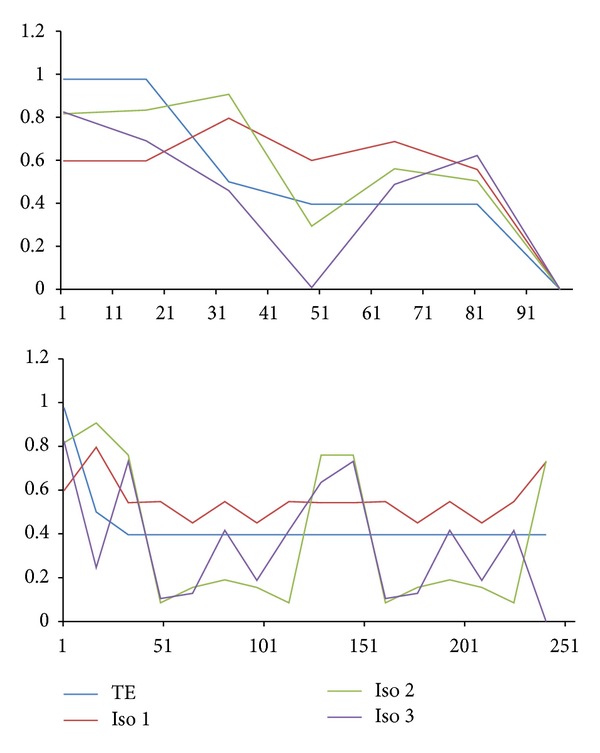
Our method compared with TE using test sequences.

**Figure 12:**
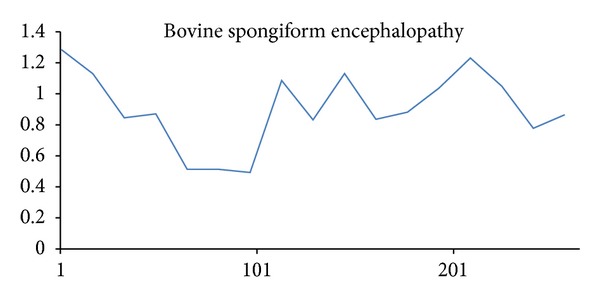
An amino acid sequence example, Bovine spongiform encephalopathy.

1. Our results are very different from those obtained by the topological entropy method; see the colored lines in Figures 11~14. These figures showed that our method is much sensitive to certain arrangements of the elements in the sequence.
2. Two different characters that exchange position will change value since Koslicki method just calculates the statistical values without structural information. Result was shown in Figure 11 bottom chart; the test sequence repeats the same subword several times. For blue line, all complexity values from topological entropy are equal within the region of repeated subwords. For red line, complexity values depend on the structure of subword. When the fragment of sequence is different from each other, our method will evaluate to different values.
3. Our method can also calculate amino acid sequences. The Koslicki method depends on alphabet size and subword size, for example, in the basic length 2 substring calculation; since standard amino acid types have up to 20, it requires a minimum length of 20^2^ + 2 − 1 to calculate, but the amino acid strings are usually very short. Sometimes, Koslicki method cannot compute the amino acid sequence efficiently.  Figure 12 shows that complexity of amino acid sequence can also be calculated by our method.

**Figure 13:**
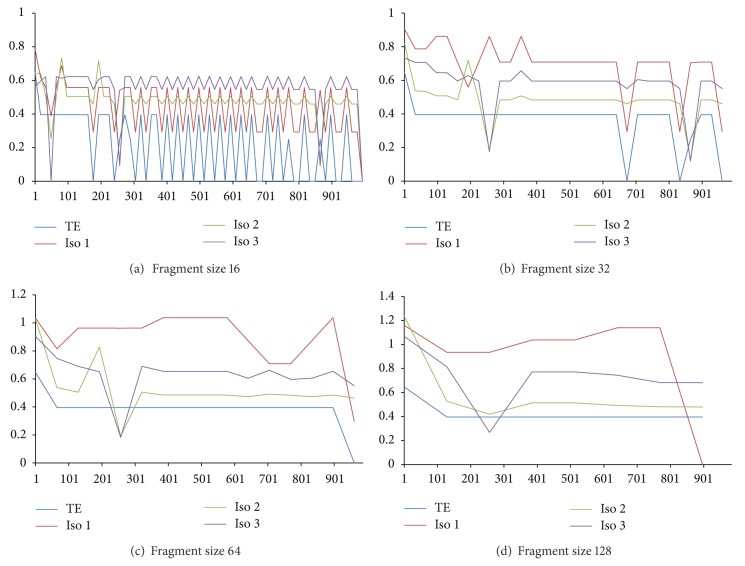
Compare with different methods.

**Figure 14:**
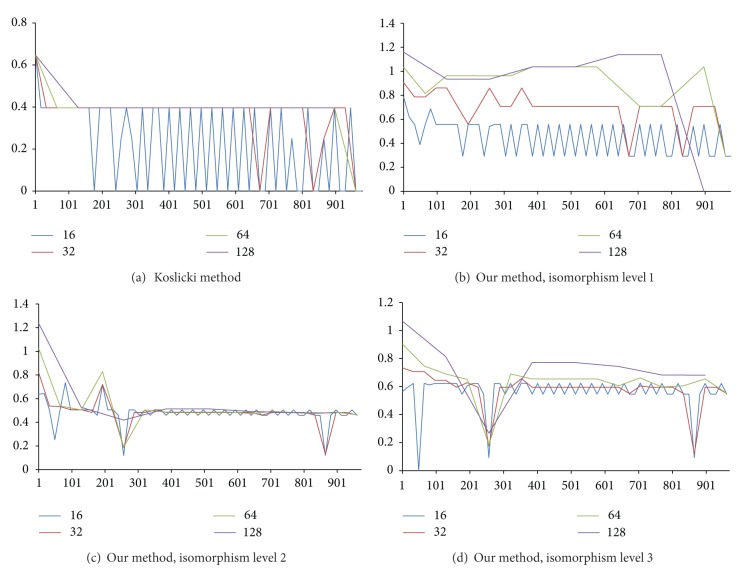
Compare with different fragment sizes.

We also did experiments with lots of data, including fixed fragment size and fixed method on test sequences (see Figures 13 and 14). Here, we redefine the Koslicki method; the fragment size is no longer dependent on subword size. Instead, fixed length fragment like our method is applied. This change allows us to compare the data easier, and not restricted to the exponentially growing fragment size anymore. In Figure 13, we found that for larger fragment, the complexity curve will become smoothly because fragments for each data point contain more information. And we note that there is a common local peak value of those figures; the *simple sequence region* is big enough that our fragment size still contains the same simple sequence.

When we compare with the same method shown in Figure 14, we found the same situation more obviously. Thus, if we have many complexity values with different sizes, we have the opportunity to restore the portion of the DNA.

### 4.1. Application to Virus Sequences Database and Other Sequences

Now we can apply our technique to Chinese word sequences. Togawa et al. [18] gave a complexity of Chinese words, but his study was based on the number of strokes, which is different from our method. Here we use Big5 encoding for our system. Since the number of Chinese words is larger than 10000, we cannot directly use words as alphabet, so we need some conversion. We read a Chinese word into four hexadecimal letters so that we can replace the sequence with tree representation and compute the complexity.

When it comes to biomedical section, we can create virus comparison database. Once a new virus or prion has been found, it will be easy to select corresponding drugs at the first time, according to cross comparison with each other by complexity in the database. We focus on most important viruses in recent years, such as *Escherichia coli* O157:H7 (*E*. *coli* o157), Enterovirus 71 (EV71), Influenza A virus subtype H1N1 (H1N1), Influenza A virus subtype H5N1 (H5N1), and severe acute respiratory syndrome (SARS). In recent years, these viruses have a significant impact and threat on the human world. We test these viruses and prions listed in Table 4. Here we can see that all prion regions cannot be analyzed by Koslicki method, but we can do it.

Finally, if any object can be written as a sequence, and there exists tree representation with alphabet of sequence, we can compute the complexity of the object.

## 5. Summary

In this paper, we give a method for computing complexity of DNA sequences. The traditional method focused on the statistical data or simply explored the structural complexity without value. In our method, we transform the DNA sequence to DNA tree with tree representations at first.

Then we transform the tree to context-free grammar format, so that it can be classified. Finally, we use redefined generating function and find the complexity values. We give a not only statistical but also structural complexity for DNA sequences, and this technique can be used in many important applications.

## Figures and Tables

**Table 1:** Rewriting rules for the DNA tree in Figure 2.

*P* → [−*FT*_*L*_][+*FT*_*R*_]
*T*_*L*_ → [−*FT*_*L*_*L*__][+*FT*_*L*_*R*__]
*T*_*L*_*L*__ → [−*FT*_*L*_*L*_*L*___][+*FT*_*L*_*L*_*R*___]
*T*_*L*_*L*_*L*___ → [−*FT*_*L*_*L*_*L*_*L*____][+*FT*_*L*_*L*_*L*_*R*____]
*T*_*L*_*L*_*L*_*L*____ → [−*F*][+*F*]
*T*_*L*_*L*_*L*_*R*____ → [−*F*][+*F*]
*T*_*L*_*L*_*R*___ → [−*FT*_*L*_*L*_*R*_*L*____][+*FT*_*L*_*L*_*R*_*R*____]
*T*_*L*_*L*_*R*_*L*____ → [−*F*][+*FT*_*L*_*L*_*R*_*L*_*R*_____]
*T*_*L*_*L*_*R*_*L*_*R*_____ → [−*F*][+*F*]
*T*_*L*_*L*_*R*_*R*____ → [−*F*][+*FT*_*L*_*L*_*R*_*R*_*R*_____]
*T*_*L*_*L*_*R*_*R*_*R*_____ → [−*F*][+*F*]
*T*_*L*_*R*__ → [−*FT*_*L*_*R*_*L*___][+*FT*_*L*_*R*_*R*___]
*T*_*L*_*R*_*L*___ → [−*FT*_*L*_*R*_*L*_*L*____][+*FT*_*L*_*R*_*L*_*R*____]
*T*_*L*_*R*_*L*_*L*____ → [−*FT*_*L*_*R*_*L*_*L*_*L*_____][+*F*]
*T*_*L*_*R*_*L*_*L*_*L*_____ → [−*F*][+*F*]
*T*_*L*_*R*_*L*_*R*____ → [−*FT*_*L*_*R*_*L*_*R*_*L*_____][+*F*]
*T*_*L*_*R*_*L*_*R*_*L*_____ → [−*F*][+*F*]
*T*_*L*_*R*_*R*___ → [−*FT*_*L*_*R*_*R*_*L*____][+*FT*_*L*_*R*_*R*_*R*____]
*T*_*L*_*R*_*R*_*L*____ → [−*FT*_*L*_*R*_*R*_*L*_*L*_____][+*FT*_*L*_*R*_*R*_*L*_*R*_____]
*T*_*L*_*R*_*R*_*L*_*L*_____ → [−*F*][+*F*]
*T*_*L*_*R*_*R*_*L*_*R*_____ → [−*F*][+*F*]
*T*_*L*_*R*_*R*_*R*____ → [−*FT*_*L*_*R*_*R*_*R*_*L*_____][+*FT*_*L*_*R*_*R*_*R*_*R*_____]
*T*_*L*_*R*_*R*_*R*_*L*_____ → [−*F*][+*F*]
*T*_*L*_*R*_*R*_*R*_*R*_____ → [−*F*][+*F*]
*T*_*R*_ → [−*FT*_*R*_*L*__][+*FT*_*R*_*R*__]
*T*_*R*_*L*__ → [−*FT*_*R*_*L*_*L*___][+*FT*_*R*_*L*_*R*___]
*T*_*R*_*L*_*L*___ → [−*FT*_*R*_*L*_*L*_*L*____][+*FT*_*R*_*L*_*L*_*R*____]
*T*_*R*_*L*_*L*_*L*____ → [−*F*][+*F*]
*T*_*R*_*L*_*L*_*R*____ → [−*FT*_*R*_*L*_*L*_*R*_*L*_____][+*F*]
*T*_*R*_*L*_*L*_*R*_*L*_____ → [−*F*][+*F*]
*T*_*R*_*L*_*R*___ → [−*FT*_*R*_*L*_*R*_*L*____][+*FT*_*R*_*L*_*R*_*R*____]
*T*_*R*_*L*_*R*_*L*____ → [−*F*][+*FT*_*R*_*L*_*R*_*L*_*R*_____]
*T*_*R*_*L*_*R*_*L*_*R*_____ → [−*F*][+*F*]
*T*_*R*_*L*_*R*_*R*____ → [−*FT*_*R*_*L*_*R*_*R*_*L*_____][+*FT*_*R*_*L*_*R*_*R*_*R*_____]
*T*_*R*_*L*_*R*_*R*_*L*_____ → [−*F*][+*F*]
*T*_*R*_*L*_*R*_*R*_*R*_____ → [−*F*][+*F*]
*T*_*R*_*R*__ → [−*FT*_*R*_*R*_*L*___][+*FT*_*R*_*R*_*R*___]
*T*_*R*_*R*_*L*___ → [−*FT*_*R*_*R*_*L*_*L*____][+*FT*_*R*_*R*_*L*_*R*____]
*T*_*R*_*R*_*L*_*L*____ → [−*FT*_*R*_*R*_*L*_*L*_*L*_____][+*F*]
*T*_*R*_*R*_*L*_*L*_*L*_____ → [−*F*][+*F*]
*T*_*R*_*R*_*L*_*R*____ → [−*F*][+*F*]
*T*_*R*_*R*_*R*___ → [−*FT*_*R*_*R*_*R*_*L*____][+*FT*_*R*_*R*_*R*_*R*____]
*T*_*R*_*R*_*R*_*L*____ → [−*FT*_*R*_*R*_*R*_*L*_*L*_____][+*FT*_*R*_*R*_*R*_*L*_*R*_____]
*T*_*R*_*R*_*R*_*L*_*L*_____ → [−*F*][+*F*]
*T*_*R*_*R*_*R*_*L*_*R*_____ → [−*F*][+*F*]
*T*_*R*_*R*_*R*_*R*____ → [−*F*][+*FT*_*R*_*R*_*R*_*R*_*R*_____]
*T*_*R*_*R*_*R*_*R*_*R*_____ → [−*F*][+*F*]

**Table 2:** Classification based on the similarity of rewriting rules.

Classification of rules	Isomorphic	Isomorphic	Isomorphic	Isomorphic
	Depth #0	Depth #1	Depth #2	Depth #3
Class #1	(19) *C*_1_ → *C*_1_ *C*_1_ (4) *C*_1_ → *C*_1_ *C*_2_ (4) *C*_1_ → *C*_2_ *C*_1_ (20) *C*_1_ → *C*_2_ *C*_2_	(8) *C*_1_ → *C*_1_ *C*_1_	(3) *C*_1_ → *C*_1_ *C*_1_	(1) *C*_1_ → *C*_1_ *C*_1_
		(1) *C*_1_ → *C*_1_ *C*_3_	(1) *C*_1_ → *C*_4_ *C*_2_	(1) *C*_1_ → *C*_4_ *C*_3_
		(1) *C*_1_ → *C*_2_ *C*_2_	(1) *C*_1_ → *C*_7_ *C*_5_	(1) *C*_1_ → *C*_5_ *C*_2_
		(1) *C*_1_ → *C*_2_ *C*_4_	(1) *C*_1_ → *C*_8_ *C*_8_	
		(1) *C*_1_ → *C*_3_ *C*_1_	(1) *C*_1_ → *C*_3_ *C*_1_	
		(1) *C*_1_ → *C*_3_ *C*_3_	(1) *C*_1_ → *C*_8_ *C*_6_	
		(1) *C*_1_ → *C*_4_ *C*_2_		
		(5) *C*_1_ → *C*_4_ *C*_4_		

Class #2	(48) *C*_2_→ null	(4) *C*_2_ → *C*_4_ *C*_5_	(1) *C*_2_ → *C*_8_ *C*_10_	(1) *C*_2_ → *C*_8_ *C*_6_
Class #3		(4) *C*_3_ → *C*_5_ *C*_4_	(1) *C*_3_ → *C*_9_ *C*_9_	(1) *C*_3_ → *C*_9_ *C*_7_
Class #4		(20) *C*_4_ → *C*_5_ *C*_5_	(1) *C*_4_ → *C*_9_ *C*_11_	(1) *C*_4_ → *C*_12_ *C*_10_
Class #5		(48) *C*_5_→ null	(1) *C*_5_ → *C*_10_ *C*_8_	(1) *C*_5_ → *C*_13_ *C*_11_
Class #6			(1) *C*_6_ → *C*_10_ *C*_10_	(1) *C*_6_ → *C*_13_ *C*_13_
Class #7			(1) *C*_7_ → *C*_11_ *C*_9_	(1) *C*_7_ → *C*_13_ *C*_15_
Class #8			(5) *C*_8_ → *C*_11_ *C*_11_	(1) *C*_8_ → *C*_14_ *C*_14_
Class #9			(4) *C*_9_ → *C*_11_ *C*_12_	(1) *C*_9_ → *C*_14_ *C*_16_
Class #10			(4) *C*_10_ → *C*_12_ *C*_11_	(1) *C*_10_ → *C*_15_ *C*_13_
Class #11			(20) *C*_11_ → *C*_12_ *C*_12_	(1) *C*_11_ → *C*_15_ *C*_15_
Class #12			(48) *C*_12_→ null	(1) *C*_12_ → *C*_16_ *C*_14_
Class #13				(5) *C*_13_ → *C*_16_ *C*_16_
Class #14				(4) *C*_14_ → *C*_16_ *C*_17_
Class #15				(4) *C*_15_ → *C*_17_ *C*_16_
Class #16				(20) *C*_16_ → *C*_17_ *C*_17_
Class #17				(48) *C*_17_→ null

**Table 3:** The values for the class parameters of Table 2.

	Classification of rules	Isomorphic depth #1
		*n*_11_ *n*_111_ *n*_112_
		(8) *C*_1_ → *C*_1_ *C*_1_
		*n*_12_ *n*_121_ *n*_122_
		(1) *C*_1_ → *C*_1_ *C*_3_
		*n*_13_ *n*_131_ *n*_132_
		(1) *C*_1_ → *C*_2_ *C*_2_
		*n*_14_ *n*_141_ *n*_142_
(*n* = 5)	Class #1 (*n*_1_ = 8)	(1) *C*_1_ → *C*_2_ *C*_4_
		*n*_15_ *n*_151_ *n*_152_
		(1) *C*_1_ → *C*_3_ *C*_1_
		*n*_16_ *n*_161_ *n*_162_
		(4) *C*_1_ → *C*_3_ *C*_3_
		*n*_17_ *n*_171_ *n*_172_
		(1) *C*_1_ → *C*_4_ *C*_2_
		*n*_18_ *n*_181_ *n*_182_
		(5) *C*_1_ → *C*_4_ *C*_4_

	Class #2 (*n*_2_ = 1)	*n*_21_ *n*_211_ *n*_212_
		(4) *C*_2_ → *C*_4_ *C*_5_
	Class #3 (*n*_3_ = 1)	*n*_31_ *n*_311_ *n*_312_
		(4) *C*_3_ → *C*_5_ *C*_4_
	Class #4 (*n*_4_ = 1)	*n*_41_ *n*_411_ *n*_412_
		(20) *C*_4_ → *C*_5_ *C*_5_
	Class #5 (*n*_5_ = 1)	*n*_51_ *n*_511_ *n*_512_
		(48) *C*_5_→ null

**Table 4:** Test data with topological entropy method and our method.

Type	Name	Koslicki method	Our method
	E. coli^a^	Available	Available
	EV71^b^	Available	Available
DNA	H1N1^c^	Available	Available
	H5N1^d^	Available	Available
	SARS^e^	Available	Available

	Abrin	Too short	Available
Amino acid	Ricin	Too short	Available
	BSE^f^	Too short	Available
	CJD^g^	Too short	Available

^a^ *Escherichia coli* O157:H7.

^b^Enterovirus 71.

^c^Influenza A virus subtype H1N1.

^d^Influenza A virus subtype H5N1.

^e^Severe acute respiratory syndrome.

^f^Bovine spongiform encephalopathy.

^g^Creutzfeldt-Jakob disease.
